# Epidemiology of dog bites to people in Uruguay (2010–2020)

**DOI:** 10.1002/vms3.1242

**Published:** 2023-08-18

**Authors:** Javier Román, Gabriela Willat, José Piaggio, María T. Correa, Juan Pablo Damián

**Affiliations:** ^1^ Facultad de Veterinaria Departamento de Ciencias Sociales Universidad de la Republica Uruguay Montevideo Uruguay; ^2^ Unidad Zoonosis y Vectores Ministerio de Salud Pública Montevideo Uruguay; ^3^ Facultad de Veterinaria Departamento de Salud Pública Veterinaria Universidad de la Republica Uruguay Montevideo Uruguay; ^4^ Department of Population Medicine and Pathobiology College of Veterinary Medicine NC State University Raleigh North Carolina; ^5^ Facultad de Veterinaria Departamento de Biociencias Veterinarias Universidad de la Republica Uruguay Montevideo Uruguay

**Keywords:** aggression, animal welfare, human–animal bond, One Health

## Abstract

**Background:**

Dog bites to people are a serious public health problem. Limited information exists at the country level in Latin America. The COVID‐19 pandemic changed people's lifestyles and their relationship with pets, and this could potentially affect the incidence of dog bites injuries.

**Objective:**

The main objectives of our study were to determine the prevalence of dog‐bite injuries in Uruguay from 2010 to 2020 and to compare the prevalence in 2020 to that of pre‐pandemic years.

**Methods:**

Cross‐sectional study. Dog‐bite notifications for the 2010 and 2020 period were analysed using data from the Uruguayan Ministry of Public Health.

**Results:**

The annual dog‐bite injury rate for the 2010–2020 period was 87.51 per 100,000 people. The frequency of bites varied with the victims’ sex, with males accounting for 51.8% of the bites (*p* < 0.0001), and with age, with a higher frequency of bites in the ≤14 years old age group (*p* < 0.01). The frequency of dog bites was also higher in spring and summer than in autumn (*p* < 0.0001). There was no statistical difference in the frequency of dog‐bite injuries when comparing 2020 with the pre‐COVID‐19 pandemic years.

**Conclusions:**

In Uruguay, the frequency of dog‐bite injuries varied with season and with the age and sex of the victim. In the first year of the COVID‐19 pandemic, the number of people bitten by dogs was no different than that of previous years. This is the first study in Latin America to report national rather than regional data and to include all age groups.

## Graphical abstract

This study describes the epidemiology of people bitten by dogs in Uruguay over a period of 11 years. The frequency of dog‐bite injuries varied with season (higher in spring and summer) and with the age (higher in people under 14 years old age) and sex of the victim (higher in males). In addition, during the first year of the COVID‐19 pandemic, the number of people bitten by dogs was no different than that of previous years.

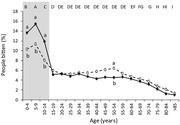

## 1 INTRODUCTION

Dog bites are a serious public health problem, resulting in numerous types of injuries and even death (Overall & Love, 2001; Quirk, 2012). They also have a negative impact on attacking dogs that may subsequently be euthanised or abandoned (Damián, 2021; Fatjó et al., 2007). The large number of dog bites resulting in human injury is an example of the One Health concept (Brookes et al., 2020; Damián, 2021).

Epidemiological data on injuries from dog bites are mainly reported from developed countries (Overall & Love, 2001; Quirk, 2012; Rosado et al., 2009; Sarcey et al., 2017). There are very few reports from Latin America and are mostly related to restricted regions or certain age groups. Benavides et al. (2019) reported injuries caused by dog bites in Brazil from 2007 to 2017, but victims’ age was not considered. Barrios et al. (2021) reported on dog bites in Chile but covered only one years´ worth of data. Comparing data on dog bites could be biased because of socioeconomic, demographic and sociocultural factors (Babazadeh et al., 2016; Barrios et al., 2021; Benavides et al., 2019; Overall & Love, 2001; Quirk, 2012; Rosado et al., 2009; Sarcey et al., 2017).

It is widely known that the pandemic (COVID‐19) caused by SAR‐COV2 affected the emotional state and mental health not only of health workers (da Silva Neto et al., 2021; Sheraton et al., 2020), but also of the general population in many different countries and continents (Kontoangelos et al., 2020; Vindegaard & Benros, 2020; Xiong et al., 2020). During the COVID‐19 pandemic, not only did the symptoms of anxiety and depression in people increase, but also the frequency of people who suffered emotional and physical violence (Hamadani et al., 2020). The changes in lifestyle and quality of life caused by the pandemic, not only affected human relationships, but also their relationship with pets. Dogs provide their owners with social support, particularly in difficult times such as the COVID‐19 pandemic, though shared activity and physical contact, among others (Bowen et al., 2020, 2021). However, during the COVID‐19 pandemic, dogs also displayed stress‐related behavioural changes (Bowen et al., 2020). During the first 3 months of the COVID‐19 pandemic, the incidence of dog bites in a Colorado children's hospital increased three times compared to pre‐pandemic years (Dixon & Mistry, 2020). The main objectives of this study were to determine the prevalence of dog bites in Uruguay from 2010 to 2020 considering season, and the age and sex of the victims, and to assess whether any differences existed pre‐ and post‐ COVID‐19 pandemic.

## 2 MATERIALS AND METHODS

In this cross‐sectional study, epidemiological data of dog bites from 2010 to 2020 were obtained from the Unit of Zoonoses and Vectors, Epidemiology Division of the Ministry of Public Health (UZV‐MSP) of Uruguay. Dog‐bite notifications are mandatory in the country for all health services, public or private. The average annual incidence (weighted mean by population density) was expressed as the number of bite incidents per 100,000 population (rate) considering the total population in Uruguay at 3,286,314 inhabitants (Official Census, INE 2011).

### 2.1 Statistical analysis

Descriptive statistics were obtained for all variables of interest.

#### 2.1.1 Effect of victim´s sex on the frequency of dog bites

The chi‐square test (*X* ^2^) of goodness of fit was used to analyse the effect of sex on the frequency of dog bites using an expected male to female ratio of 50:50.

#### 2.1.2 Comparison of the age when males or females are bitten

The age (continuous variable) of people bitten between both sexes was compared using the Mann–Whitney *U* test, since it did not have a normal distribution, and data were expressed as medians (± 95% CI).

#### 2.1.3 Influence of the season of the year, age categories and sex of the victim

People bitten by dogs were classified into sex (male or female), according to the season of the year in which they were bitten (summer, autumn, winter and spring), and into 18 age categories (0–4, 5–9, 10–14, 15–19, 20–24, 25–29, 30–34, 35–39, 40–44, 45 −49, 50–54, 55–59, 60–64, 65–69, 70–74, 75–79, 80–85, >85 years). The percentage of people bitten by dogs by age category or by season within each sex and year for the overall study was analysed via a mixed‐effect analysis of variance model (SAS Studio, SAS OnDemand for Academics). The fixed effects were age category and sex, and an interaction between sex and age was tested using GLIMMIX procedures with a lognormal distribution. In a second model, fixed effects for season and sex and an interaction term between them were included and analysed using the GLM procedure with a normal distribution. Post hoc comparisons were made with the Tukey‐Kramer test. Significant differences were considered at an alpha ≤ 0.05.

#### 2.1.4 Influence of the COVID‐19 pandemic

Categorical variables were analysed using the chi‐square test to determine associations between sex with dog bites by pre‐ and post‐pandemic years (2010 to 2019 vs. 2020). A contingency table was used, and the odds ratio (OR) was calculated and expressed with its the 95% CI. The age at which people were bitten pre‐ and post‐pandemic (2010 to 2019 vs. 2020) was compared using the Mann–Whitney *U* test, and results were expressed as medians (± 95% CI).

## 3 RESULTS

The prevalence of dog bites is shown in Table 1. There were 31,634 notifications of dog bites between 2010 and 2020, which represents an annual rate of 87.51 injuries per 100,000 people in Uruguay (Table 1).

**TABLE 1:** People bitten by dogs in Uruguay per year from 2010 to 2020.

Year	*n*	%	Rate (95% CI)
2010	2578	8.15	78.45 (76.52–80.38)
2011	2401	7.59	73.06 (69.99–76.13)
2012	1830	5.78	55.69 (48.93–62.45)
2013	3309	10.46	100.69 (97.89–103.49)
2014	3416	10.80	103.95 (100.46–107.44)
2015	3164	10.00	96.28 (94.41–98.14)
2016	2796	8.84	85.08 (84.56–85.59)
2017	3064	9.69	93.24 (92.02–94.45)
2018	3231	10.21	98.32 (96.02–100.61)
2019	3332	10.53	101.39 (98.44–104.33)
2020	2513	7.94	76.47 (74.12–78.81)
2010–2020	31,634	100	87.51

*n*: number of dog bites; %: percentages of dog bites; Rate: injury rates are per 100,000 population; 95% CI: confidence interval.

### 3.1 Effect of victim´s sex on the frequency of dog bites

The frequency of dog bites varied with the victim´s sex (*p* < 0.0001), the frequency of males (*n* = 16,336, 51.8%) being greater than that of females (*n* = 15,189, 48.2%) when considered over the entire study period.

### 3.2 Comparison of the age when males or females are bitten

The median age of male injuries was 23.0 (±1.0 95% CI) compared with a median age of 33.0 (±1.0 95% CI) for females (*p* < 0.0001).

### 3.3 Influence of the season of the year, age and sex of the victim

**FIGURE 1:**
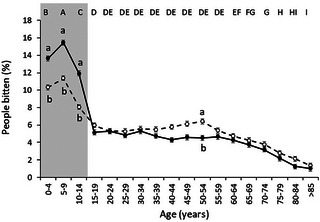
Dog bites in Uruguay during the period 2010 to 2020. Percentage of people bitten (mean ± SEM) by age categories and sex (male: ‐●‐ or female: ‐○‐). Different capital letters between age categories represent points that differ significantly (*p* < 0.05). Different lowercase letters between male and female for the same age categories represent points that differ significantly (*p* < 0.0001).

Sex distribution was highly dependent on age (*p* < 0.0001), with a predominance of males over females in the <15‐year age groups (*p* < 0.0001) (Figure 1). In the >25‐year age categories, there was a tendency for females to predominate with this reaching significance in the 50–54 age group (*p* = 0.0056). Overall, dog bites were much more frequent in the youngest age categories (under 14 years old) (*p* < 0.01).

**FIGURE 2:**
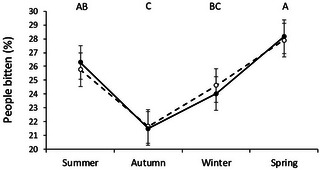
Dog bites in Uruguay during the period 2010 to 2020. Percentage of people bitten (mean ± SEM) by season of the year and sex (male: ‐●‐ or female: ‐○‐). Different capital letters between seasons of the year represent points that differ significantly (*p* < 0.0001).

The percentage of dog bites varied by season of the year (*p* < 0.0001), with most bites occurring in spring and summer, and fewest in winter (Figure 2). Male to female ratio was not significantly different between the seasons (*p* = 0.99).

### 3.4 Influence of the COVID‐19 pandemic

As shown in Table 1, the number of dog bites in the year of the pandemic (2020) was similar to that of previous years.

There was however an association between the pandemic and the sex (*p* = 0.015; OR = 1.106 [95% CI: 1.020 to 1.200]), the predominance of males versus females in the years prior to the pandemic (52% vs. 48%) being reversed in the year of the pandemic (49.5% vs. 50.5%).

The age of the victims in the year of the pandemic (median 26.0 ± 1.0 95% CI) was also lower than that of previous years (median age: 28.0 ± 1.0 95% CI) (*p* < 0.0001).

## 4 DISCUSSION

This is the first epidemiological study of dog bites in Uruguay covering reports from the entire territory and all age categories. The biting prevalence in Uruguay is lower than that reported in other countries from North America (Daigle et al., 2022; Overall & Love, 2001; Quirk, 2012) and from Oceania (Mair et al., 2019). Studies from Asia have variably reported higher (Abedi et al., 2019; Amiri et al., 2020; Babazadeh et al., 2016; Gongal & Wright, 2011) and slightly lower prevalence (Masiira et al., 2018; Pal et al., 2021). Similarly, the frequencies reported in European countries have been both higher (Westgarth et al., 2018) and slightly lower (Rosado et al., 2009) than found in our study. In relation to data from the region, in Uruguay, the rate of bites/100,000 inhabitants is somewhere between that of Chile and Brazil. In Uruguay, it is mandatory to report dog bites although there is possible underreporting if treatment is done at home, something that has been reported in other countries (Overall & Love, 2001; Quirk, 2012). It is evident that there is a great variability in the rate of dog bites between countries and between continents. Based on the results of this study we suggest that, on a global level, Uruguay occupies an intermediate place, confirming that dog‐bite injuries represent an important public health problem in this country.

In accordance with reports from other countries such as Spain, France, the United States and Chile (Barrios et al., 2021; Overall & Love, 2001; Quirk, 2012; Rosado et al., 2009; Sarcey et al., 2017), the under 14 years of age group received most bites. The behaviour of children, lack of understanding of dogs’ body language prior to the aggression and lack of adult supervision are some of the reasons that may explain the higher incidence of bites in this age group (Overall & Love, 2001; Quirk, 2012). Indeed, Lakestani et al. (2014) reported that children interpret dog behaviour differently than adults. These data reinforce the need to implement educational campaigns and programs at the school level, so that children and adolescents can better understand and interpret animal behaviour, more specifically dogs.

Considering all age groups, males were bitten more often than females, a finding that has also been reported in other countries (Amiri et al., 2020; Masiira et al., 2018; Overall & Love, 2001; Quirk, 2012; Rosado et al., 2009). In the under 14 years of age group, this male predominance was even more pronounced, and this has also been reported previously (Overall & Love, 2001; Quirk, 2012; Rosado et al., 2009). In fact, the predominance of males in the under 14 age group, and their increased risk of bite injury, is likely to account for the increased frequency of bites in males of all ages. In the early stages of growth, male children are more active and have more energetic movements and attitudes, which could trigger more dog attacks (Mathews & Lattal, 1994). This pattern was reversed at older ages when females were affected in larger proportions. These results coincide with other studies like the one from Chile, which found that the highest frequency of dog bites for females was for the 40 and over age group (Barrios et al., 2021). Taken as a whole, the current epidemiological information shows that, in adults, dog‐bite numbers vary according to sex in different countries and/or continents. These gender differences could be explained by cultural differences in people´s behaviour towards and exposure to dogs (Mathews & Lattal, 1994; Overall & Love, 2001; Rosado et al., 2009). In Uruguay there are no studies on the human–dog bond but, in other countries, it has been observed that females have more interaction and emotional closeness, and a greater attachment and propensity to be hoarders of dogs than males (Fatjó et al., 2013; Patronek & Nathanson, 2009). Given that the interaction between humans and dogs depends on cultural and demographic factors, future studies are necessary to investigate the social context in which such aggressive events occur in Latin American countries.

Another interesting finding was that the percentage of people bitten by dogs was higher in the spring and summer. A Spanish study by Rosado et al. (2009), and one in the United States by Overall and Love (2001), also found the highest frequency of bites in the summer. It is likely that the warmest months of the year coincide with vacations and outdoor activities. However, despite the proximity of the two countries, our result differs from those obtained in Chile by Barrios et al. (2021), who reported the highest frequency of bite incidents in winter and autumn. The reason for these differences remains unclear but it highlights that, even within the same continent, data should not be extrapolated from one country to another.

During the first year (2020) of the health emergency caused by the COVID‐19 pandemic, the number of people bitten by dogs in Uruguay was similar to that of previous non‐pandemic years. This differs from the results reported by Dixon and Mistry (2020) and Tulloch et al. (2021) who found a marked increase in the incidence of dog bites during the first months of the COVID‐19 pandemic in the United States and the United Kingdom, respectively. During the first year of the COVID‐19 pandemic, the level of free movement of people in Uruguay was reduced but the degree of quarantine and the level of isolation was not as extreme as in many other countries. In addition, the start of the pandemic and corresponding movement control measures began at the beginning of autumn, which coincides with the lowest incidence of dog bites. This may have attenuated its effects on the incidence of dog bites. Regardless, it is clear that, in addition to cultural, social and economic differences, different countries have chosen different ways of responding to the COVID‐19 pandemic, resulting in different impacts on the relationship between people and their respective pets.

Interestingly, although the number of dog bites during the pandemic was within the range of previous years, the prevalence by sex and age varied slightly during the pandemic. In the year of the pandemic, dog bites were more frequent in the females, and the median age was lower than in previous years. However, these associations were weak and, considering the data as a whole, the year of the pandemic did not appear to affect the prevalence of dog bites in Uruguay.

Considering our data, strategies to help prevent bite injuries at a country level should include educational prevention programs for children in schools, awareness campaigns to promote wider knowledge of dog behaviour and interpretation of corporal signals, legislation to ensure adequate supervision and responsible dog ownership and early instigation of puppy socialisation and obedience training. These strategies go hand in hand with reinforcement of the concept of ‘One Health’ and strengthening of collaborative work between doctors and veterinarians within the community (de Keuster & Overall, 2011; Gilchrist et al., 2008; Meints & de Keuster, 2009; Overall, 2010).

In conclusion, dog‐bite injuries represent an important public health problem in Uruguay, with a rate of 87.51/100,000 inhabitants. The Uruguayan population mostly affected were male and children under 14 years of age, and most bites occurred in the spring and summer. During the first year (2020) of the health emergency caused by the COVID‐19 pandemic, the number of people bitten by dogs was similar to that of previous years.

## AUTHOR CONTRIBUTIONS

Javier Román: data curation; investigation; writing – original draft. Gabriela Willat: project administration; resources. José Piaggio: validation; visualisation. María T. Correa: formal analysis; methodology; validation; visualisation; writing – original draft; writing – review & editing. Juan Pablo Damián: conceptualisation; data curation; formal analysis; investigation; methodology; resources; software; supervision; validation; visualisation; writing – original draft; writing – review & editing.

## CONFLICT OF INTEREST STATEMENT

The authors declare that there is no conflict of interest.

## ETHICS STATEMENT

No ethical approval was required for the study. It did not involve human subjects or animals, and the data used were publicly available.

### PEER REVIEW

The peer review history for this article is available at https://www.webofscience.com/api/gateway/wos/peer‐review/10.1002/vms3.1242.

## Data Availability

The data that support the findings of this study are available from the corresponding author upon reasonable request.
